# Oral–Gut Microbiota Crosstalk and Epigenetic Targets in Metabolic and Neuropsychiatric Diseases

**DOI:** 10.3390/nu17213367

**Published:** 2025-10-27

**Authors:** Sahar Mostafavi, Shabnam Nohesara, Ahmad Pirani, Hamid Mostafavi Abdolmaleky, Sam Thiagalingam

**Affiliations:** 1 Department of Orthodontics and Dentofacial Orthopedics, Tufts University School of Dental Medicine, Boston, MA 02111, USA; sahar.mostafavi@tufts.edu; 2 Department of Medicine (Biomedical Genetics), Boston University Chobanian and Avedisian School of Medicine, Boston, MA 02118, USA; snohesar@bu.edu; 3 Mental Health Research Center, Psychosocial Health Research Institute, Iran University of Medical Sciences, Tehran 14535, Iran; 4 Department of Medicine, Beth Israel Deaconess Medical Center, Harvard Medical School, Boston, MA 02215, USA; 5 Department of Pathology and Laboratory Medicine, Boston University Chobanian and Avedisian School of Medicine, Boston, MA 02118, USA

**Keywords:** oral microbiota, neuropsychiatric disorders, metabolic abnormalities, epigenetic, nutrition, inflammation

## Abstract

The oral cavity contains a diverse group of bacteria in the saliva, as well as structured aggregates of bacterial cells on the mucosal surfaces. Oral microbiota (OM) dysbiosis not only induces local inflammation, it can also trigger systemic inflammation leading to metabolic diseases and neuropsychiatric diseases (NPDs). While primary evidence indicates that oral microbiota dysbiosis induces gut microbiota aberrations, which exacerbate inflammation associated with metabolic diseases (obesity, dyslipidemia, diabetes, nonalcoholic fatty liver disease (NAFLD), and insulin resistance), other studies revealed the contribution of the oral microbiota–brain axis in the pathogenesis of NPDs. GM dysbiosis and inflammation also induce epigenetic alterations in cytokine genes, such as IL-1β, IL-6, TNF-α, NF-kB, BTLA, IL-18R1, TGF-β, P13k/Akt1, Ctnnb1, and Hsp90aa1, as well as DNMTs, HDACs, and DAT1 associated with the development and progression of metabolic disorders and/or NPDs. Therefore, the epigenome could serve as a target for preventive or therapeutic interventions. Here, we (i) review emerging evidence of the potential impact of OM dysbiosis in the pathogenesis of metabolic diseases and NPDs, (ii) highlight the relationship between OM-induced inflammation and epigenetic alterations driving NPDs pathogenesis and interlinked metabolic aberrations, (iii) discuss therapeutic approaches capable of treating metabolic diseases and NPDs through reshaping the microbiota and its epigenetic metabolites, and hence mitigating epigenetic aberrations linked to metabolic diseases and NPDs. Finally, we outline challenges and current research gaps related to investigating the relationship between microbiota, epigenetic aberrations, and metabolic abnormalities associated with NPDs.

## 1. Introduction

Metabolic disorders and neuropsychiatric disorders (NPDs) are wide-ranging classes of diseases, each with a complex nature and high degree of genetic heterogeneity that negatively influence various body organs, the nervous system, and mental health conditions like mood, thinking, cognition, and behavior [1,2,3,4]. Common NPDs include several categories of disorders, such as neurodevelopmental (e.g., autism spectrum disorders (ASDs)), neurodegenerative (e.g., Parkinson’s disease (PD) and Alzheimer’s disease (AD)), and psychiatric disorders (e.g., bipolar disorder (BD) and schizophrenia (SCZ)) [5,6]. Metabolic disorders are conditions that influence any aspect of metabolism and are associated with energy balance dysregulation [7]. Examples of metabolic disorders are obesity, dyslipidemia, diabetes, metabolic syndrome, nonalcoholic fatty liver disease (NAFLD), and insulin resistance [8]. Notably, as will be discussed in this work, most NPDs are associated with metabolic diseases. Therefore, NPDs and metabolic diseases are interlinked and may, at least in part, originate from a common underlying functional aberration [9,10].

Epigenetic alterations have been found to be one of the key players in the pathogenesis of both metabolic diseases and NPDs by defining how environmental factors such as infections, malnutrition, and stress could interplay with genes to affect the function of different organs and contribute to the development of various diseases [11,12,13]. Abnormalities in epigenetic modifications in DNA methylation, histone modifications, and non-coding RNAs (ncRNAs) contribute to disease susceptibility by influencing gene expression without changing the DNA sequence itself [14]. Interestingly, while epigenetic modifications influence cellular metabolism, the metabolic state of cells can also induce epigenetic changes that, in turn, affect cellular functions, including those of brain cells, and thereby impact mental health [15,16]. Multiple lines of experimental evidence have suggested an interesting link between NPDs and metabolic dysregulation, which may cause epigenetic modifications [17,18]. For example, AD is considered type 3 diabetes [19]. Subjects with BD and SCZ also exhibit an elevated risk for metabolic disturbances, OB, and metabolic syndrome, which may be associated with epigenetic aberrations [16,20]. Approximately 37% of subjects with BD demonstrate symptoms of metabolic syndrome, which contribute to worsening of the course of BD, leading to both poor treatment response and disability [21]. Some of the metabolic pathways contribute to one-carbon metabolism (involving folic acid, betaine, choline, methionine, and vitamins B6 and B12), which determines DNA and histone methylation levels, playing key roles in brain function [22]. Derangements in the functionality of responsible metabolic pathways may contribute to the development of metabolic diseases and NPDs due to, or associated with, epigenetic aberrations [23]. For example, Frajerman et al. examined the prevalence of folate and vitamin B12 deficiency and hyperhomocysteinemia in subjects with psychotic disorders ranging in age from 15 to 30 years [24]. Their findings showed deficiencies in folate, vitamin B12 (key products of some bacteria of gastrointestinal (GI) tracts), and hyperhomocysteinemia (one of the consequences of folate and vitamin B12 deficiency) in 38% of subjects with first-episode psychosis, 27% of schizophrenic subjects, and 36% of patients with schizoaffective disorders [24]. Therefore, while extensive epigenetic aberrations are reported in different NPDs [25,26], metabolic dysregulations due to an imbalanced nutritional conformation and/or oral and gut bacteria composition affecting the body reserve of these key vitamins may be linked to the development or worsening of NPDs through epigenetic mechanisms [27].

The mouth is the main artery for the entry of environmental microbiota to the GI tract. The oral cavity is a complex environment, and its microorganisms inhabit several distinctive niches such as the gingival sulcus, the tongue, saliva, teeth, the hard and soft palates, the cheek, the floor of the mouth, and the throat [28,29]. Experimental evidence indicates that while oral microbiota (OM) is affected by metabolic diseases such as type 2 diabetes (T2DM) [30], it can affect the brain and mental health through the oral–brain axis. This axis is bidirectional and mediates a complex interplay between oral microbes, the metabolic, immune, and nervous systems, and influences the function of neuronal cells through the activity of specific microorganisms [31]. In pathological conditions such as periodontitis, imbalance in the OM composition causes dysbiosis, which contributes to systemic inflammation and the release of specific cytokines and neurotoxins, contributing to the development of metabolic diseases and NPDs [32,33,34]. Oral bacteria are capable of producing different metabolites that affect brain function by passing the blood–brain barrier. Moreover, pathogenic microbes in the oral cavity are capable of entering the bloodstream, initiating inflammatory responses, and even translocating to the brain via the trigeminal nerve or olfactory system [27]. Abnormal alterations in the composition and diversity of OM are also associated with an increased risk and severity of metabolic diseases and NPDs. For example, increase in the abundance of oral pathogens, like *Porphyromonas gingivalis* (*P. gingivalis*), in combination with virulence factors like lipopolysaccharides (LPS) and gingipains cause neuroinflammation, which in turn leads to cognitive decline [35]. On the other hand, Alex et al. found that women with recent excessive life stresses and symptoms of depression exhibited an increased abundance of *Proteobacteria* and *Spirochaetes*, respectively, in saliva samples [36]. Additionally, increased abundance of the members of the phylum Firmicutes was seen in pregnant women with high levels of anxiety and depressive symptoms [36]. Lee et al. also found an increased abundance of pathogenic taxa, such as *Veillonella* and *Prevotella*, in the OM of patients with SCZ-related psychosis and psychotic BD [37].

In addition to microbially induced neuroinflammation, NPD pathogenesis (the process by which a disease or disorder develops) may be linked to abnormal metabolic and epigenetic changes involving metabolites such as short-chain fatty acids (SCFAs) synthesized by oral and gut microbes via two other processes, including carbohydrate hydrolysis or amino acid metabolism [38,39].

**Figure 1:**
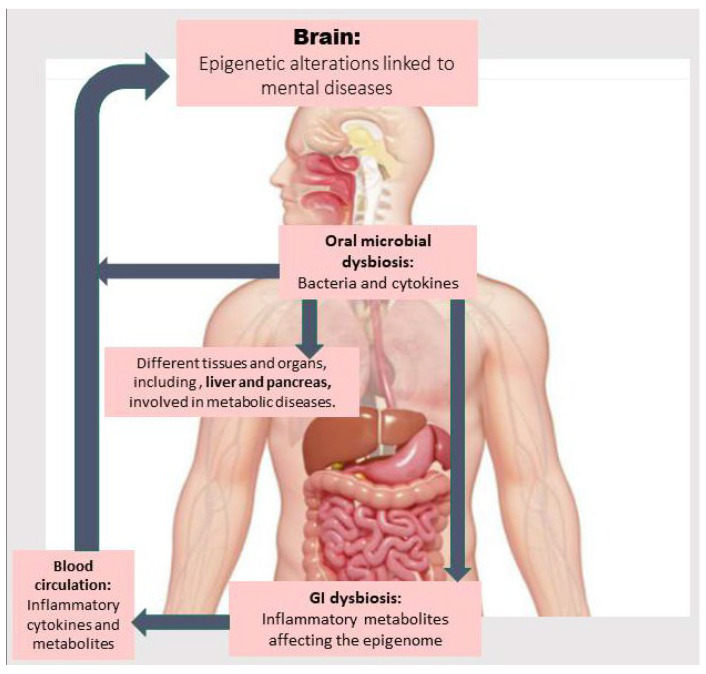
The oral microbiota, by passing through the oropharynx, the sole gateway to the gastrointestinal (GI) tract, influences the gut microbiome. Oral microbial dysbiosis is associated not only with the invasion of bacteria and inflammatory cytokines into the bloodstream, but it can also induce GI dysbiosis. This leads to the production of toxic and inflammatory metabolites that trigger cytokine production by white blood cells, which induce epigenetic alterations in the brain or in organs related to metabolic diseases (e.g., the liver), which in turn can affect brain function.

In this article, we first review the existing literature about the relationship between oral dysbiosis and developing metabolic diseases and NPDs. Then, we discuss how abnormalities in metabolic pathways and those related to one-carbon metabolism (involved in methylation reactions) due to oral and gut dysbiosis may contribute to inflammation and the onset and development of metabolic diseases and NPDs via epigenetic aberrations. Next, we explore the main findings about therapeutic approaches capable of treating metabolic diseases and NPDs by modulating the OM, improving metabolic abnormalities through reshaping the gut microbiota (GM) and its epigenetic metabolites, and mitigating epigenetic aberrations. Finally, we discuss challenges and current research gaps related to investigating the correlation between OM, epigenetic aberrations, and metabolic abnormalities associated with NPDs and the GM along with corresponding preventive and therapeutic interventions. Figure 1 provides an overview of how OM dysbiosis may directly introduce bacterial elements and inflammatory cytokines into the bloodstream, as well as induce gut dysbiosis, thereby contributing to epigenetic alterations linked to the pathogenesis of metabolic diseases and related NPDs. These mechanisms are described in detail in the following sections.

## 2. Oral Microbiota and the Pathogenesis of Metabolic Diseases

It has been shown that oral dysbiosis is linked to the development of metabolic diseases, which in turn contribute to the onset and aggravation of periodontal disease and tooth loss. Several lines of evidence have shown an interesting link between metabolic disease (obesity, dyslipidemia, and metabolic syndrome) and periodontitis [40,41,42]. Obesity is linked to tooth loss five years later, and the periodontal condition of individuals with obesity is inferior to that of subjects with normal weight [43]. Obesity is capable of altering both the OM and GM composition and, hence, contributing to the development of oral diseases via inflammation. For example, leptin-deficient obesity in mice is associated with a decreased abundance of beneficial bacteria, including *Akkermansia* and *Ruminococcaceae_UCG_014*, and increased abundance of inflammation-related *Flavobacterium* in the salivary samples, indicating that leptin-deficient obesity is a risk factor for developing periodontitis [44]. Sato et al. found that obesity elevates the risk of periodontal disease by escalating production of uric acid, mediated by gut dysbiosis [45]. Jia et al. reported that subjects classed as obese exhibited the highest proportion of severe periodontitis (stage III and IV) and higher amounts of the inflammatory mediators in gingival crevicular fluid versus controls [46]. On the other hand, periodontitis and oral dysbiosis may contribute to the development of metabolic diseases. For example, salivary microbiota of subjects with periodontitis are capable of worsening liver function in HFD-induced obese mice, and contribute to the development of NAFLD by disrupting gut barrier function, activating the TLR4 signaling pathway, and causing liver inflammation [47]. Here, first, we will review the emerging evidence of the potential impact of OM dysbiosis in the pathogenesis of metabolic diseases and NPDs (Table 1).

It is also worth noting that the association between metabolic diseases and periodontitis may be mediated by epigenetic mechanisms. For example, the DNA methylation status of buccal cells may represent markers related to obesity and metabolic disorders [58]. As another example, individuals with obesity and periodontitis exhibited overexpression of miR-200b in gingival tissue [59]. Byun et al. also found that salivary exosomal miR-25-3p plays a key role in developing and aggravating diabetes-related periodontitis [60]. Liu et al. reported that miR-223 and miR-200b in the gingival crevicular fluid are strongly connected to the pathogenesis of the periodontal disease and vulnerability to T2DM [61]. In another study, elevated levels of miR-146a were observed in T2DM compared to non-diabetic, periodontally healthy subjects [62]. A different clinical study also reported that miRNA 146a is a reliable marker of periodontitis among diabetic patients, with an optimum cut-off value of ≥11.04 and an accuracy of 86.1% [63].

## 3. Oral Microbiome and the Pathogenesis of NPDs

The oral microflora is a key player in oral health and a contributor to systemic health by influencing host physiological mechanisms. People with greater oral microbial α-diversity exhibit better cognitive performance status [64]. In an interesting study, Qiao et al. examined the potential role of mouth–microbial–brain connections in the development of NPDs and found that the OM of ASD children could cause ASD-like behaviors by changing microbial community structures and neural signaling activities in the prefrontal cortex of recipient mice, along with the upregulation of genes related to serotonin and TGF-β signaling pathways [65]. As ASD children exhibit lower oral bacterial diversity versus controls [66], the lower OM α-diversity is linked to a greater risk of depression as well [67]. In PD, aberrant salivary OM releases harmful metabolites that pass through biofilm and trigger iron dysregulation, collectively perturbing the function of the mouth–gut–brain axis and leading to degenerative damage and necrosis of dopamine neurons [68]. Furthermore, while increased intestinal permeability and elevated serum LPS-binding protein levels have been found in subjects newly diagnosed with PD [69], higher levels of specific gut microbial genera, such as *Cloacibacterium*, *Microbacterium*, and *Isoptericola*, have been detected in the blood samples of patients with PD [70]. Periodontal pathogens may also induce or accelerate the progression of AD via the formation of beta-amyloid protein (Aβ) and, subsequently, increasing neuroinflammation and other pathogenic pathways [71]. In addition, as OM dysbiosis is connected to a greater brain Aβ load and the onset or progression of AD, oral-derived microbes, such as *P. gingivalis*, have been detected in the brains of patients with AD [72]. However, small-molecule inhibitors targeting gingipains, the neurotoxic components of *P. gingivalis*, can reduce brain bacterial load following *P. gingivalis* infection, decrease Aβ1–42 production and neuroinflammation, and ultimately rescue neurons in the mouse hippocampus [73]. Mechanistically, *P. gingivalis* infection in iPSC-derived neurons has been shown to increase autophagic vacuoles and multivesicular bodies, the phospho-tau/tau ratio, synapse loss, and cytoskeletal disruption [74]. A growing body of studies has also shown a correlation between psychosocial factors and periodontitis. Wu et al. found that adolescents with BD exhibited an elevated risk for periodontitis [75]. Moreover, depression is considered a risk factor for periodontal disease [76]. Therefore, not only can OM aberrations predispose individuals to metabolic and mental diseases, but NPDs are also associated with a higher risk of periodontitis, which in turn could exacerbate NPDs, creating a vicious cycle. Other studies addressing the correlation between changes in OM composition and the pathogenesis of different types of NPDs are summarized in Table 2.

## 4. OM-Induced Inflammation Drives Metabolic and Epigenetic Alterations Underlying NPD Pathogenesis

**Figure 2:**
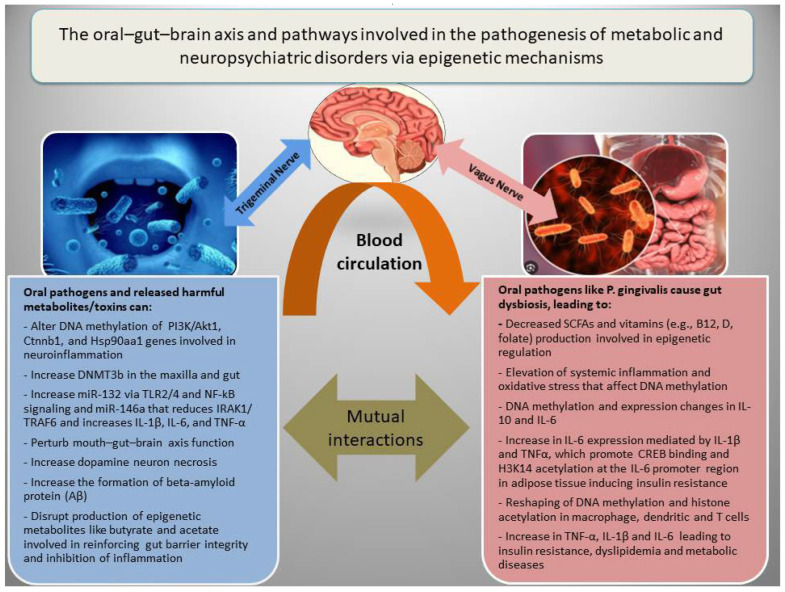
The oral–gut–brain axis, inflammation, and epigenetic alterations in metabolic and neuropsychiatric disorders. Oral pathogens can promote gut dysbiosis and cooperatively induce inflammation, disrupt gut barrier integrity, and impair the function of the oral–gut–brain axis by affecting the production of short-chain fatty acids (SCFAs) such as butyrate and acetate, as well as vitamins involved in epigenetic modifications in both the brain and metabolic organs.

In addition to genetic factors, the pathogenesis of NPDs is associated with epigenetic dysregulation of metabolic and inflammatory genes, which interact with diverse socioeconomic, dietary, ecological, and seasonal conditions [18,20,88]. There are three main epigenetic mechanisms that regulate gene expression, including (a) DNA methylation, which in general inhibits gene expression; (b) histone modifications, which may increase or decrease gene expression depending on the nature of the modification; and (c) RNA interference, which involves miRNAs and lncRNAs, generally associated with the inhibition of gene expression or increased RNA degradation [12]. Epigenetic modifications have been among the major mechanisms for adaptation to environmental changes or fluctuations during ordinary life and throughout evolution. However, adversary environmental factors such as malnutrition, chronic infections, and oral or gut microbial dysbiosis may lead to epigenetic alterations and cause metabolic or mental diseases by inducing oxidative stress and inflammation [89]. Figure 2 shows how the oral–gut–brain axis and its related pathways are involved in the pathogenesis of metabolic diseases and NPDs via epigenetic mechanisms.

For instance, experimental studies revealed that orally administered *P. gingivalis* could induce periodontitis and differentially methylated regions related to *PI3K/Akt1*, *Ctnnb1*, and *Hsp90aa1* genes involved in inflammation (TNF signaling) and NPDs, in multiple tissues in mice [90]. However, treatment with an anti-TNF-α antibody could mitigate the host response to *P. gingivalis* and reduce serum TNF-α, IL-6, blood glucose levels, and the size of the *P. gingivalis* inoculation lesion in a mouse model for type 2 diabetes and obesity [91]. Experimental periodontitis and *P. gingivalis* gavage could also induce expression of inflammatory markers (BTLA and IL-18R1) and increase DNMT3b, a marker of de novo DNA methylation in the gut and maxilla of C57BL/6 mice [92]. With respect to the causal link between inflammation and epigenetic aberrations, Niwa T and Ushijima reviewed how chronic inflammation (e.g., gastritis and hepatitis) triggers aberrant DNA methylation even in normal tissues. They pinpoint mechanisms like cytokine-stimulated cell proliferation and ROS-driven DNA damage that recruit DNMTs (DNA methyl transferases) to specific gene promoters, creating an “epigenetic field for cancerization” [93]. Another comprehensive review highlighted how inflammatory stimuli (such as LPS and cytokines IFN-γ) or IL-4, an anti-inflammatory cytokine, reshape DNA methylation and histone acetylation in macrophages, dendritic cells, and T cells, which reprogram gene expression during innate memory and T cell polarization [94]. A meta-analysis on epigenome-wide association studies also identified 58 CpG sites whose methylation levels in blood correlate with CRP (a chronic inflammation marker) level, and many of these methylation alterations are linked to cardiometabolic disease [95]. Rodríguez-Ubreva et al. showed that monocytes from septic patients, and even healthy monocytes exposed to LPS (TLR4 stimulus), undergo significant DNA methylation changes associated with IL-10 and IL-6 level alterations [96].

Beyond DNA methylation changes, oral dysbiosis in vivo and exposure of oral epithelial cells to LPS in vitro induce histone modifications, activate the transcriptional coactivators p300/CBP, and promote NF-κB accumulation [97]. Additionally, a clinical study analyzing saliva samples reported downregulation of the histone deacetylase genes *HDAC4*, *HDAC8*, and *HDAC10* in gingivitis, and *HDAC4*, *HDAC6*, *HDAC8*, and *HDAC9* in periodontitis [98]. Remarkably, six months of non-surgical periodontal therapy not only improved clinical periodontal parameters but also upregulated the expression of *HDAC2*, *HDAC4*, *HDAC6*, *HDAC8*, *HDAC9*, and *HDAC11* in saliva, indicating a causal relationship [99]. Other studies revealed that a shorter duration of such therapy (3 months) could also decrease the levels of MMP-8 (matrix metalloproteinase-8), MAF (macrophage-activating factors), and SIRT1 (an NAD+-dependent histone deacetylase) in saliva, while increasing the serum level of T-SOD (total superoxide dismutase, an antioxidant enzyme) in patients with periodontitis [100].

Inflammatory oral bacteria can affect miRNA expression as well. For example, *P. gingivalis* infection in THP-1 macrophages upregulates miR-132 via TLR2/4 and NF-κB signaling. Gingival fibroblasts and macrophages exposed to *P. gingivalis* LPS also show significant upregulation of miR-146a (and 146a-5p), which reduces *IRAK1/TRAF6* expression and increases IL-1β, IL-6, and TNF-α secretion [101]. Similarly, in vivo polymicrobial periodontal infection can induce miR-146a in both local periodontal tissues and the spleen of ApoE^−^/^−^ mice [102]. Experimental evidence also supports that alterations in the OM composition in NPDs, such as ASDs, may be associated with epigenetic alterations [103]. For example, children with ASDs exhibit miRNA and microbiota dysregulations in the saliva, which are connected to their cognitive impairments [104]. Ragusa et al. found an association between changes in specific miRNA expression (e.g., upregulation of miR-29a-3p and miR-141-3p and downregulation of miR-16-5p, let-7b-5p, and miR-451a) and OM composition in children with ASDs (e.g., elevated levels of *Actinobacillus*, *Weeksellaceae*, *Rothia*, *Filifactor*, *Ralstonia*, *Pasteurellaceae*, and *Aggregatibacter* and reduced levels of *Tannerella*, *Moryella*, and the *TM7-3* bacterial group) [104]. Their findings showed a negative relationship between salivary miR-141-3p expression and *Tannerella* abundance in saliva, which was linked to cognitive dysfunction [104].

With respect to metabolic dysfunction, large-scale metagenomic analysis of OM compositional changes in NPDs (such as ASDs) revealed significant alterations in metabolic pathways involved in the degradation of serotonin, GABA, and dopamine; key players in brain function and psychopathogenesis [85]. Reciprocally, impaired dopamine signaling in ASDs is also associated with OM alterations, alongside metabolic and gastrointestinal dysfunction. For example, the ASD-associated variant in the *SLC6A3* gene (dopamine transporter, also known as *DAT1*) leads to a significant reduction in *Fusobacterium* abundance in the DAT T356M^+/+^ mouse oral cavity, where increased *Fusobacterium* abundance is linked to improved glucose handling and reduced body fat [105]. In the saliva of patients with bulimia nervosa and binge-eating disorder, the abundance of specific OM genera (e.g., increased abundance of *Bacilli* and depletion of *Lachnospirales*) is linked to exosomal miRNA expression changes (e.g., upregulation of let-7b-5p, mir-15b-5p, mir-429, and mir-221-3p), and DNA hypomethylation of the *DAT1* gene [106]. Altogether, these studies provide experimental evidence that OM composition influences inflammatory cytokines and the epigenetic setting related to the pathogenesis of NPDs and their metabolic counterparts. Table 3 presents an overview of the mechanisms by which oral microbiota dysbiosis, through the induction of epigenetic aberrations, may contribute to inflammation and thereby increase the risk of developing metabolic diseases and NPDs.

In other mental diseases such as SCZ, patients not only exhibit significant changes in the OM composition, including elevated levels of specific genera such as *Neisseria* and *Porphyromonas*, but also display upregulation of key metabolic pathways such as β-alanine metabolism and vitamin digestion and absorption [107]. The findings of this study suggest that elevated levels of certain metabolites, such as L-methionine sulfoxide (L-MetO) and tyramine, resulting from oral dysbiosis, may contribute to the initiation of oxidative stress in SCZ patients [107]. Oxidative stress, in turn, is a well-known driver of inflammation, as well as metabolic and epigenetic alterations [18]. In AD, François et al. reported that disease progression was associated with alterations in oral bacterial composition, including a decreased abundance of *Lautropia mirabilis* and remarkable changes in vitamin B12 metabolism, and reduced levels of salivary Transcobalamin-1, which binds and protects vitamin B12 (a cofactor in methylation reactions) from degradation by stomach acid [108]. Additionally, while several studies have shown that the disruption of the microbial profiles in the oral cavity is linked to altered functionality of metabolic pathways, it has been shown that the oropharynx microbial profile correlates with the OM profile both in normal conditions and in children with ASDs [109,110]. Since the oropharynx is the exclusive gateway to the GI tract, OM can be considered as the main mediator of the gut microbial composition; it is a well-known contributor to the pathogenesis of NPDs, inflammation, and metabolic diseases [2,110,111]. In line with this, a recent experimental study concluded that “oral-to-gut translocation may be the main route” of environmental microbial translocations to the GI tract [112]. Via this route, the OM is not only directly linked to the pathogenesis of metabolic and mental diseases, but is also indirectly involved in shaping the GM and local or systemic inflammation. For instance, experimental data show that in patients with periodontitis, the salivary microbiota can induce gut dysbiosis because swallowing of the pathogenic OM can perturb the GM composition [113]. Furthermore, Kitamoto et al. experimentally induced periodontitis in a mouse model and found that oral pathobionts can migrate to the colon, significantly worsening inflammation in DSS-induced colitis. The mechanism involved Th17 T cells, initially “primed in oral mucosa–draining lymph nodes”, then trafficked to the gut and reactivated by oral microbes, driving elevated IL-1β and colonic pathology [114]. Research data also support that a dysbiotic OM in periodontitis can trigger excessive secretion of IL-17A by innate and adaptive immune cells; this cytokine fosters both local periodontal destruction and systemic inflammatory diseases [115], and contributes to neuroinflammation, as well as the dysregulation of a wide range of neurotransmitters and neuromodulators, increasing the risk of NPDs [116]. It has been shown that, at least in an AD mouse model, IL-17A induces promoter DNA methylation of the *Bmal1* (brain and muscle ARNT-like 1) gene, decreasing its expression and leading to disruption of the circadian rhythm. In this process, the MAPK pathway mediates IL-17A-induced *Dnmt1* upregulation, which in turn leads to DNA methylation of the *Bmal1* promoter [117].

Based on other animal experiments, oral inoculation with *P. gingivalis* may also lead to macrophage infiltration into adipose tissue and elevate systemic inflammatory markers, increasing insulin resistance, likely via the oral–gut axis influencing gut barrier function and the microbiome [118]. Periodontal pathogens (e.g., *P. gingivalis*) can also enter the bloodstream and cause bacteremia/endotoxemia. This stimulates endothelial cells and immune components, triggering systemic cytokine rises (TNF-α, IL-1β, and IL-6), thereby contributing to atherosclerosis, insulin resistance, hypertension, and dyslipidemia [119]. Another study uncovered that while adipose tissue IL-6 is the main contributor to insulin resistance, IL-1β and TNF-α cooperativity increase *IL-6* expression by promoting CREB binding and H3K14 acetylation at the *IL-6* promoter region [120]. Additionally, a review of several original studies linked oral dysbiosis in periodontitis with autoimmune mechanisms—via pathways like Toll-like receptors, molecular mimicry, and bystander activation—potentially contributing to diseases like rheumatoid arthritis and systemic inflammation beyond the oral cavity [121]. Therefore, from a preventive or therapeutic point of view, while preserving oral health and its microbiota composition is important to prevent inflammation, it is also critical to reshape OM-induced GM alterations to mitigate epigenetic alterations associated with metabolic and mental diseases. The focus of the following section is the use of prebiotics or probiotics (i.e., nutritional agents that modulate oral or GM composition) to prevent or treat OM-induced metabolic and/or associated mental diseases.

## 5. Therapeutic Remedies Based on Modulation of Oral Microbiome

Given the link between OM and GM with metabolic diseases and NPDs involving epigenetic modifications, in addition to oral hygiene, therapeutic strategies could include microbiota-targeted and nutritional interventions to help mitigate metabolic and epigenetic aberrations in NPDs.

### 5.1. Oral Hygiene to Prevent or Improve OM-Induced Metabolic Dysfunctions and NPDs

Lack of an adequate hygiene routine and reducing salivary flow due to dehydration or use of different medications, including psychoactive substances or prescribed drugs with anticholinergic side effects (e.g., most traditional antidepressants and antipsychotics), and difficulty in accessing dental health services may increase the growth rate and activity of periodontopathogenic bacteria. As an example, *P. gingivalis* and *T. denticola* as well as their toxic proteases (gingipains) have been identified in postmortem brain analyses of AD patients, with the extent of tau and ubiquitin pathology found to correlate with their levels. Additional mechanistic studies revealed that oral infection with *P. gingivalis* in mice led to brain colonization, increased levels of the amyloid plaque component Aβ1–42, and neurotoxic effects of gingipains on the tau protein. Treatment with a small-molecule gingipain inhibitor reduced brain bacterial load, blocked Aβ1–42 production, decreased neuroinflammation, and prevented hippocampal neuronal loss, indicating a causal role of gingipains in both neuroinflammation and neuronal loss [73]. Improving oral health and oral hygiene practice may be a promising approach to attenuate inflammation during metabolic diseases [122]. For example, frequent tooth brushing decreases the risk of hypertension and T2DM, as brushing at least twice a day may help prevent future occurrences of these conditions [123].

Some oral pathogens are linked to vitamin B deficiency and, hence, the development of various diseases. For instance, Avcu et al. found that subjects with poor Oral Hygiene Index (OHI) scores had the most frequent gastric recurrence of *H pylori* (58.3%) versus those with fair OHI scores (41.2%) and good OHI scores (4.8%) [124]. Their results also showed that eradication of *H pylori* in dental plaque could contribute to recovery from anemia with increases in the serum vitamin B12 level [124]. In specific metabolic diseases such as diabetes, which is much more common in patients with major mental diseases such as SCZ and BD [122], oral hygiene has a more critical role in general health status. For example, the relative abundance of *P. gingivalis* is higher in patients with T2DM [30]. The relative abundance of *Firmicutes*, a pathogenic bacterium, is also higher in diabetic patients with advanced periodontitis [125]. Another recent study reported higher salivary abundance of *Firmicutes* in adult diabetic patients with periodontitis as well [126]. Periodontal health problems in diabetic patients with chronic hyperglycemia could induce oral microbial dysbiosis and provoke pathological pathways, such as inflammation, and oxidative stress leading to periodontal tissue damage and interlinked systemic diseases [127]. Altogether, these studies suggest that oral microbial dysbiosis and related dental and gingival diseases not only pose a risk to general health and contribute to gut dysbiosis, but that systemic diseases—particularly metabolic disorders—may also impact oral health, potentially creating a vicious cycle that worsens both conditions. Therefore, the prevention and treatment of periodontal diseases require a systemic approach, including the management of gut dysbiosis.

### 5.2. Nutritional Interventions and Pre-, Pro-, and Postbiotics to Improve Oral Health and OM-Induced Epigenetic Diseases, Metabolic Diseases, and NPDs

Owing to the critical roles of some dietary factors’ involvement in microbial dysbiosis and dental or gingival erosions, nutritional interventions may contribute to improving oral health and, subsequently, preventing or treating metabolic diseases and related NPDs. For example, the low pH and high titratable acidity of some soft drinks and the metabolizing of their sugar by plaque microorganisms, hence producing organic acids, can cause dental erosion [128]. Vitamin deficiency may accelerate several non-specific oral conditions like glossitis, stomatitis, and mucosal ulceration [129]. As an example, an early sign of vitamin B12 deficiency is glossitis with linear lesions [130]. It appears that correcting disruptions in one-carbon metabolism through the use of various B vitamins may help prevent metabolic diseases and NPDs by supporting a healthy oral or gut microbiota and enhancing the growth and activity of commensal bacteria [131]. Almost all of the B vitamins, such as folate, vitamin B12, vitamin B6, and riboflavin (vitamin B2), are involved in methylation reactions as coenzymes and, hence, their supplementation may contribute to improving NPDs via modulation of the microbiota composition and mitigating epigenetic and metabolic dysregulations [132]. In an interesting study, Wang et al. found that treatment of rats exposed to chronic unpredictable mild stress by the vitamin B complex could prevent homocysteine-induced derangements in DNA methylation and, subsequently, reduce stress-related cognitive decline [133]. Holmes et al. also found that increased levels of homocysteine in subjects with mild cognitive impairment were linked to elevated rates of epigenetic aging, and treatment with B vitamins could hamper accelerated epigenetic aging [134]. Folic acid may also be considered as an adjuvant treatment in patients with periodontal disease [135], as well as in SCZ patients with significant improvement in their core symptoms, as shown in a randomized control trial study [136]. Additionally, adequate intake of folate, vitamin B6, and vitamin B12 is associated with better cognitive performance—as demonstrated in a longitudinal study of patients with MCI—by regulating DNA methylation [137,138]. Considering other vitamins, a recent systematic review and meta-analysis of 16 studies, including nearly 18,000 individuals, concluded that higher vitamin C intake is linked to an almost 50% lower risk of periodontal disease [139]. In another meta-analysis of 11 studies, while circulating vitamin C level correlated inversely (~40% reduced risk) with metabolic syndrome [140], its deficiency was linked to depression and cognitive impairment [141]. One of the main underlying mechanisms of these correlations is epigenetic alterations, since vitamin C is a powerful epigenetic modulator that activates TET enzymes, leading to DNA demethylation at the regulatory regions of genes [142]. Vitamin D deficiency is also linked to periodontitis. For example, a meta-analysis of 16 studies revealed that a lower serum vitamin D level was associated with a higher risk of periodontitis [143]. Another meta-analysis of 23 studies reported that a higher vitamin D level was linked to an almost 20% reduced risk of metabolic syndrome [144]; other meta-analyses have reported that its deficiency is associated with a higher risk of dementia (32%) [145], depression (40%) [146], and SCZ [147]. Interestingly, while Jiang et al. reported that the deficiency of vitamin D metabolites in school-age children with obesity affects DNA methylation of metabolic and vitamin D metabolism genes [148], an animal study revealed that maternal vitamin D deficiency can induce epigenome alterations in multiple generations [149]. Altogether, these data suggest that the deficiency of vitamins linked to dental health is also linked to metabolic and mental diseases, mediated by epigenetic changes bridging the connection between oral health and metabolic and/or mental diseases.

## 6. Critical Points, Limitations, and Future Perspectives

Critical points regarding OM-induced inflammation triggering epigenetic alterations that drive NPD pathogenesis and interlink metabolic aberrations include the complex interplay between microbial dysbiosis, host immune responses, and epigenetic regulation. Although numerous associations or correlative studies indicate that OM dysbiosis can induce chronic inflammation, potentially altering epigenetic marks in neural and metabolic pathways and contributing to disease development, significant shortcomings remain. Many studies are associative, lacking causal evidence, and most are conducted in preclinical models, which may not fully translate to human physiology [150,151]. Therefore, although several meta-analyses of human studies support the link between OM and metabolic or epigenetic alterations in NPDs, our understanding of the precise molecular mechanisms by which specific oral bacteria influence metabolic or epigenetic changes, as well as the temporal relationship between inflammation and epigenetic remodeling, is still limited. Additional pitfalls include the variability in individual microbiomes, environmental factors, and host genetics, all of which complicate the reproducibility and generalizability of findings. There is also a lack of standardized biomarkers to reliably track metabolic or epigenetic changes in OM-induced inflammation in clinical settings. These gaps hinder the development of targeted therapies and early diagnostics. Future studies need to integrate multi-omics approaches and longitudinal designs to elucidate causal pathways and identify precise therapeutic targets.

Efforts to improve oral hygiene as a way to prevent or mitigate OM-induced metabolic dysfunction and NPDs are grounded in evidence linking OM dysbiosis to systemic inflammation and the pathogenesis of diverse diseases; however, critical shortcomings must be acknowledged. First, interventions like tooth-brushing and mouth rinses, while reducing oral pathogens, may only provide transient improvements in microbial balance, especially if host or environmental factors (e.g., systemic diseases, diet, food preservatives, or contaminants) drive recurring dysbiosis. Second, most current studies are cross-sectional or correlative, which limits definitive conclusions about causality, meaning oral hygiene alone may be insufficient to significantly reduce the risk or severity of metabolic or mental disorders. Finally, individualized host responses and genetic variations influence intervention efficacy, which remains an underexplored area. Therefore, a comprehensive approach is required to deal with these conditions.

Nutritional interventions, including dietary modifications and the use of vitamins, pre-, pro-, and postbiotics, offer a promising approach to improve both oral health and OM-induced epigenetic, metabolic, and neuropsychiatric outcomes. Probiotics (like *Lactobacillus* and *Bifidobacterium*) can modulate the oral microbiome, suppress pathogens, reduce inflammation, and improve local and systemic immunity [152]. Nevertheless, challenges exist as the stability and colonization ability of probiotics in the oral environment vary by strain and may be affected by factors such as diet and host immunity. There is also wide variability in dosing, formulation, and treatment duration, and beneficial effects may be temporary if supplementation stops. Additionally, there are potential risks related to overuse or inappropriate combinations of biotic interventions, such as microbial resistance, unintended dysbiosis, or negative immune reactions.

Future research should prioritize large-scale, longitudinal, and interventional studies to clarify the mechanistic links between OM and disease pathogenesis as well as preventive or therapeutic approaches such as oral hygiene, targeted nutritional interventions, and the prevention or mitigation of systemic inflammation, metabolic dysfunction, and NPDs. Once causal links are established between OM dysbiosis, or specific oral bacteria, and metabolic diseases or NPDs, the development of personalized, microbiome-based therapies tailored to individual microbial profiles, seasonal and ecological variations, and host genetics holds great promise for maximizing potential efficacy and minimizing adverse effects. More robust and harmonized methodologies in clinical trials, such as standardizing intervention protocols and outcome measures, are essential to advance the field and allow replication across diverse populations. Innovations such as next-generation probiotics, synbiotics (prebiotic and probiotic combinations), and postbiotics (beneficial microbial metabolites, such as butyrate and acetate) merit further exploration, as do non-invasive biomarkers to monitor response and guide adaptive treatment [153]. Ultimately, integrating oral health into a broader framework of preventive and precision medicine could substantially impact the management of metabolic and neuropsychiatric disorders linked to the oral microbiome.

## 7. Conclusions

In this article, we reviewed the potential mechanistic links between OM, epigenetic dysregulations, and the pathogenesis of NPDs and metabolic diseases. We propose that oral and gut dysbiosis may contribute to the development of metabolic diseases and NPDs by disrupting metabolic pathways involved in epigenetic regulation. Therefore, simultaneous targeting of metabolic and epigenetic dysregulations using some specific diets, probiotics, and prebiotics may be considered as potential therapeutic approaches for the prevention or treatment of NPDs and metabolic diseases. The concept of the oral–brain axis is an area of interest with ever-increasing growth that may contribute to the prevention or treatment of NPDs, and may contribute to easier diagnosis by providing new microbial signatures and epigenetic marks. Future studies should focus on developing novel therapeutic approaches that simultaneously target metabolic and epigenetic dysregulations, building upon a profound understanding of the potential mechanistic links between NPD pathogeneses and metabolic/epigenetic abnormalities, to achieve more effective treatment of metabolic diseases and NPDs. This field remains largely unexplored, and further investigations with larger sample sizes of unmedicated patients are needed to examine the biological links and interconnections between the OM, GM, and various classes of metabolic diseases and NPDs in order to establish consistent patterns and obtain more definitive findings. Certainly, technological advances in measurement techniques and more precise classifications of the oral and gut microbiota, together with their biological products and effects, will also help identify key disease-specific pathogenic bacterial species and susceptible individuals, thereby informing therapeutic strategies.

## Figures and Tables

**Table 1:** Association between metabolic diseases and alterations in the OM composition.

Metabolic Disorder	Type of Study/Number of Participants	Microbiota Testing Method/Relevant Pathway or Oral Disease	Key Findings and Affected Oral Bacteria	Ref.
Obesity	Human/9 healthy normal weight and 10 classed as obese	16S rRNA gene sequening/periodontal inflammation	Higher abundance of the *Capnocytophaga* genus (2.47% vs. 0.27%) in subjects classed as obese vs. normal weight	[48]
Obesity	Human/34 healthy normal weight and 11 classed as obese	16S rRNA gene sequening/periodontal inflammation	Reduction in OM diversity and an elevation in periodontal inflammation; greater abundance of *Staphylococcaceae* (1.13% vs. 0.48%) in periodontal microbiota in subjects classed as obese vs. normal weight	[49]
Obesity	Human/25 classed as obese or overweight vs. 25 of normal weight	16S rRNA gene sequencing/moderate–severe periodontitis	Increased abundance of periopathogens, including *Aggregatibacter* and *actinomycetemcomitans* in subjects with obesity	[50]
Type 2 diabetes (T2DM)	Human/280 patients with T2DM and 162 healthy controls	16S rRNA gene sequencing/increased risk of periodontitis	Increased *Neisseria* (14.12%), *Streptococcus*, *Haemophilus*, and *Pseudomonas* genera, decreased *Acinetobacteria*, and elevated Firmicutes/Bacteroidetes ratio (7.6% vs. 2.74%) in T2DM vs. controls	[51]
T2DM	Human/15 patients with T2DM vs. controls	16S rRNA gene sequencing/inflammation	Reduced saliva *Fusobacteriota* and *Campilobacterota* and increased *Proteobacteria* abundance in T2DM, triggering the NLRP3 inflammasome pathway	[52]
T2DM	Human/183 patients with type 2 diabetes and 74 controls	16S rRNA gene sequencing/inflammatory mediators of oral and intestinal flora	Higher oral *Streptococcus*, *Actinobacteria*, *Rothia*, *Cetobacterium*, and intestinal *Bifidobacterium*, *Streptococcus*, and *Blautia* correlate with T2DM; upregulated glycine betaine degradation pathway in oral and intestinal flora	[53]
T2DM	Human/10 patients with type 2 diabetes and 10 controls	Metagenomic sequencing/OM dysbiosis in T2DM and increased risk of oral diseases	Increased periodontal pathogens like *P. gingivalis* and *Prevotella melaninogenica* and harmful salivary metabolites such as cadaverine and L-(+)-leucine	[54]
Metabolic syndrome (MetS)	Human/128 subgingival plaque samples from participants with and without MetS	Metagenomic and 16S rRNA gene sequencing/inflammation	Increased abundance of *Actinomyces dentalis*, *Actinomyces naeslundii*, *Actinomyces viscosus*, *Corynebacterium matruchotii*, *Leptotrichia buccalis*, and *Streptococcus sanguinis* in MetS	[55]
Metabolic-associated fatty liver disease (MAFLD)	Human/24 patients with MAFLD and 22 healthy controls	16S rRNA gene sequencing/chronic low-grade inflammation	Increased *Actinomyces* and *Prevotella* 2 spp. in supragingival plaques in MAFLD; insulin resistance correlates with the abundance of *Granulicatella*, *Veillonella*, *Streptococcus*, and *Scardovia* spp., and obesity with *Streptococcus*, *Olsenella*, *Scardovia*, and *Selenomonas* spp.	[56]
Dyslipidemia	Human/763 tongue coating samples	16S rRNA gene sequencing/lipid metabolism	Increased relative abundance of *Megasphaera* in dyslipidemia; dyslipidemia is linked to the abundance of *Veillonella*, *Atopobium*, *Stomatobaculum*, *Tanneralla*, and *Megasphaera*	[57]

**Table 2:** Altered oral microbiota (OM) composition in neuropsychiatric diseases.

Neuropsychiatric Disorders	Study Type/Samples/Microbiota Testing Method	Oral Microbiota Changes in Patients	Effects on the Disease State	Ref.
Schizophrenia (SCZ) and mania	Human/throat swab/16S rRNA gene sequencing	Decreased *Neisseria subflava*, *Weeksellaceae*, and *Prevotella* and increased *Streptococci*	Correlation between *Neisseria subflava* and cognitive performance	[77]
SCZ	Human/swabs from the middle site of the tongue/Illumina MiSeq sequencing	Decreased *Prevotella* and *Veillonella*; increased *Streptococcus* and *Fusobacterium*	OM composition affects peripheral inflammatory cytokines	[78]
SCZ	Human/saliva/16S rRNA gene sequencing	Increased in some Gram-positive (*Actinomyces*, *Rothia*, *Atopobium*, *Streptococcus*) and Gram-negative bacteria (*Prevotella*, *Leptotrichia Porphyromonas*, *Lautropia*, and *Capnocytophaga*)	OM dysbiosis is associated with brain functional connectivity changes	[79]
SCZ	Human/saliva/16S rRNA gene sequencing	Decreased *Actinobacteriota* in patients	A poor oral environment is associated with altered OM in SCZ	[80]
Alzheimer’s disease (AD)	Human/saliva and periodontal samples/16S rRNA gene sequencing	*Streptococcus oralis* and *Porphyromonas gingivalis* are the predominant salivary and periodontal bacteria, respectively	Association between altered OM and cognition in AD	[81]
AD vs. cognitively unimpaired patients with periodontitis	Human/buccal, supragingival, and subgingival plaque samples/16S rRNA gene sequencing	Increased *Atopobium rimae*, *Dialister pneumosintes*, *Olsenella* sp. *HMT 807*, *Saccharibacteria (TM7)* sp*. HMT 348*, and several species of *Prevotella* in AD	Direct association between periodontitis caused by OM dysbiosis and greater cognitive decline	[82]
AD	Human/saliva/16S rRNA gene sequencing	Increased *Fusobacteriota* and *Peptostreptococcaceae* and decreased *Veillonella* vs. the MCI and controls	Association between abnormal immune responses and inflammatory processes and OM changes in AD	[83]
Parkinson’s disease (PD)	Human/saliva/shotgun metatranscriptomic profiling	Decrease in a bacteriophage (*Streptococcus* phage PhiSpn 200), and increase in three yeast species (*Candida albicans*, *Candida dubliniensis*, and *Saccharomyces cerevisiae*) in PD	Significant alterations in several indices of motor, cognitive, and sensory function	[84]
Autism spectrum disorders (ASDs)	Human/saliva/metagenomic approach	Increased *Actinomyces hongkongensis*, *Actinomyces johnsonii*, *Cutibacterium acnes*, the *Eikenella* species NML 130454, and *Rothia dentocariosa*	Association between the OM composition and cognitive impairments in ASDs	[85]
Depression	Human/saliva/16S rRNA gene sequencing	Increased *Neisseria* spp. and *Prevotella nigrescens*	OM dysbiosis is associated with depression	[86]
Depression	Human/saliva/16S rRNA gene sequencing	Decreased *Neisseria* genus	Negative correlation between *Neisseria* genus and pro-inflammatory cytokines	[87]

**Table 3:** Experimental evidence of OM-dysbiosis-induced epigenetic modifications and inflammatory responses.

Experiment	Epigenetic Changes	Immunoassays/Outcome	Ref.
Orally administered *P. gingivalis* in mice to induce periodontitis	Methylation of *PI3K/Akt1*, *Ctnnb1*, and *Hsp90aa1*	ELISA/increases inflammation (TNF signaling), which is reversed by TNF*-α* inhibition	[90,91]
*P. gingivalis* gavage in mice, which induces periodontitis	Increases *DNMT3b* expression, likely due to “repercussion”	Immunofluorescence staining and Procarta Multiplex Cytokine Kit/increases inflammatory markers BTLA and IL-18R1	[92]
Monocytes exposed to LPS or monocytes of patients with sepsis	DNA methylation changes related to inflammatory genes	The cytometric bead array and ELISA/alterations in IL-10 and IL-6 levels	[96]
Oral dysbiosis in vivo and oral epithelial cells exposure to LPS in vitro	Induces histone modifications	Immunohistochemistry, immunofluorescence, and immunoblotting/activates the transcriptional coactivators p300/CBP, and promotes NF-κB accumulation	[97]
Clinical gingivitis	Downregulates *HDAC4*, *HDAC8*, and *HDAC10* in saliva in gingivitis, and *HDAC4*, *HDAC6*, *HDAC8*, and *HDAC9* in periodontitis	ELISA plate reader/3–6 months non-surgical periodontal therapy improved HDACs changes; decreased salivary MMP-8 and MAF (inflammatory) and increased serum T-SOD (antioxidant) levels	[98,99,100]
*P. gingivalis* infection in THP-1 macrophages or *P. gingivalis* LPS	Upregulates miR-132 via TLR2/4 and NF-κB signaling and miR-146a	RT-PCR and ELISA/reduces *IRAK1/TRAF6* expression and increases IL-1β, IL-6, and TNF-α secretion	[101]
In vivo polymicrobial periodontal infection in ApoE^−^/^−^ mice	Induces miR-146a in periodontal tissues and the spleen	RT-PCR/reduces *IRAK1/TRAF6* expression and increases IL-1β, IL-6, and TNF-α secretion [101]	[102]
*Tannerella* abundance in saliva in ASD	Increases salivary miR-141-3p expression	Increases cognitive dysfunction	[104]
Increased salivary *Bacilli* and reduced *Lachnospirales in eating disorder*	Upregulation of exosomal let-7b-5p, mir-15b-5p, mir-429, and mir-221-3p, and hypomethylation of *DAT1*	RT-PCR/increases dopamine transporter (*DAT1*) gene expression affecting dopamine signaling	[106]

## Data Availability

Not applicable.
